# P91 Time to care: a comparative evaluation of IV versus oral antibiotic administration

**DOI:** 10.1093/jacamr/dlaf230.098

**Published:** 2025-12-04

**Authors:** Yasmine Johnstone, Euan Proud, Elaine Sharp

**Affiliations:** NHS Forth Valley, Larbert, Scotland; NHS Forth Valley, Larbert, Scotland; NHS Forth Valley, Larbert, Scotland

## Abstract

**Background:**

Healthcare is a significant contributor to global emissions, with the sector accounting for large proportion of emissions worldwide. The NHS aims to achieve net-zero carbon emissions, necessitating constant evaluation of clinical practices that contribute to environmental waste. A key antimicrobial stewardship goal; IV to oral switch encompasses these key areas, with oral antibiotics presenting a potentially cheaper, time-efficient and less environmentally damaging option while maintaining effectiveness.

**Objectives:**

The aim of this evaluation was to compare the nursing time taken for preparation and administration of IV antibiotics versus oral antibiotics and to assess and quantify the associated clinical waste and potential environmental impact. A further aim was to compare the cost between preparations.

**Methods:**

A literature review was conducted to inform project design and contextualize findings. A data collection sheet was developed to record antibiotic type, route, preparation time, administration time and waste materials used. Observations were conducted over three days on a surgical ward within a district general hospital. Nursing staff were timed using a stopwatch during their preparation and administration of antibiotics. Equipment use was categorized (e.g. syringes, infusion bags, PPE) and quantified for each observed dose.

**Results:**

The data collected included 36 IV preparations, 28 IV administrations and 18 oral administrations. The average time required to prepare and administer IV antibiotics was 14.12 min, compared to 0.27 min for oral antibiotics, a 98.1% difference. Over one month, this equated to 295.81 h of nursing time for IV antibiotic tasks, versus 5.66 h if all doses were oral. Waste analysis showed IV antibiotics generated significant single-use plastic waste, including multiple syringes, water for injection vials, giving sets and PPE per dose. Flucloxacillin produced the highest volume of waste due to its high dosing. In contrast, oral antibiotics generated minimal waste. The associated cost was considerably higher per IV dose when compared to their oral equivalent.

**Conclusions:**

A substantially greater impact on nursing time was observed with IV antibiotic use versus oral. Oral antibiotics were additionally observed to cost less and produce minimal waste, when compared with IV, particularly where high daily doses were required. These findings are relevant given the existing workload pressures faced by nursing staff, where reducing time spent on IV preparation and administration could free capacity for direct patient care. Furthermore, the results highlight the importance of adopting more sustainable antibiotic prescribing practices. One solution is to reduce IV antibiotic burden by implementing prompt IVOST. Further research is needed to explore the wider impact across health boards and support national policies aligned with NHS sustainability goals.

